# Validity and reliability of GPS and LPS for measuring distances covered and sprint mechanical properties in team sports

**DOI:** 10.1371/journal.pone.0192708

**Published:** 2018-02-08

**Authors:** Matthias W. Hoppe, Christian Baumgart, Ted Polglaze, Jürgen Freiwald

**Affiliations:** 1 University of Wuppertal, Department of Movement and Training Science, Wuppertal, Germany; 2 University of Western Australia, Exercise and Sport Science, School of Human Sciences, Perth, Australia; Universita degli Studi di Verona, ITALY

## Abstract

This study aimed to investigate the validity and reliability of global (GPS) and local (LPS) positioning systems for measuring distances covered and sprint mechanical properties in team sports. Here, we evaluated two recently released 18 Hz GPS and 20 Hz LPS technologies together with one established 10 Hz GPS technology. Six male athletes (age: 27±2 years; VO_2_max: 48.8±4.7 ml/min/kg) performed outdoors on 10 trials of a team sport-specific circuit that was equipped with double-light timing gates. The circuit included various walking, jogging, and sprinting sections that were performed either in straight-lines or with changes of direction. During the circuit, athletes wore two devices of each positioning system. From the reported and filtered velocity data, the distances covered and sprint mechanical properties (i.e., the theoretical maximal horizontal velocity, force, and power output) were computed. The sprint mechanical properties were modeled via an inverse dynamic approach applied to the center of mass. The validity was determined by comparing the measured and criterion data via the typical error of estimate (TEE), whereas the reliability was examined by comparing the two devices of each technology (i.e., the between-device reliability) via the coefficient of variation (CV). Outliers due to measurement errors were statistically identified and excluded from validity and reliability analyses. The 18 Hz GPS showed better validity and reliability for determining the distances covered (TEE: 1.6–8.0%; CV: 1.1–5.1%) and sprint mechanical properties (TEE: 4.5–14.3%; CV: 3.1–7.5%) than the 10 Hz GPS (TEE: 3.0–12.9%; CV: 2.5–13.0% and TEE: 4.1–23.1%; CV: 3.3–20.0%). However, the 20 Hz LPS demonstrated superior validity and reliability overall (TEE: 1.0–6.0%; CV: 0.7–5.0% and TEE: 2.1–9.2%; CV: 1.6–7.3%). For the 10 Hz GPS, 18 Hz GPS, and 20 Hz LPS, the relative loss of data sets due to measurement errors was 10.0%, 20.0%, and 15.8%, respectively. This study shows that 18 Hz GPS has enhanced validity and reliability for determining movement patterns in team sports compared to 10 Hz GPS, whereas 20 Hz LPS had superior validity and reliability overall. However, compared to 10 Hz GPS, 18 Hz GPS and 20 Hz LPS technologies had more outliers due to measurement errors, which limits their practical applications at this time.

## Introduction

In recent years, the tracking of team sport players has switched away from camera-based technology, which requires manual player labeling [1] and has problems with overlapping players, dress colors, and changes in light and shadow conditions [2]. As a replacement, global (GPS) [3] and also local (LPS) [4] positioning systems have become standard tools for determining movement patterns during matches and training sessions [5, 6]. While investigations of matches are useful to establish performance profiles and provide a framework to design training and testing procedures, the analysis of training sessions permits a periodization on a daily basis [6]. However, to allow a meaningful interpretation of GPS and LPS data for the aforementioned practical applications, knowledge of the validity and reliability of the technologies for measuring the data of interest is essential [7]. In this context, previous studies have focused on distances covered and velocity measurements (e.g., instantaneous, mean, or peak) using known distances combined with timing gates [6, 8] and theodolites [9], motion capture system [4, 10], or laser/radar [2, 11] derived velocities as the criterion measure.

Presently, there is one review that summarizes the evidence from the many studies that evaluated available GPS devices operating at 1–15 Hz for determining movement patterns in team sports [12]. Overall, 1–15 Hz devices all permit a valid and reliable determination of the total distance covered. However, low sample rate 1–5 Hz devices have limitations for measuring distances covered during high velocity runs, accelerated runs over short distances, and velocity measures, especially when changes of direction (CODs) are performed. While 10 Hz devices overcome most of these limitations, 15 Hz devices show no superior validity and reliability [12]. Notably, the 15 Hz devices investigated [10, 13–15] merely up-sample a 5 Hz signal [14]. Consequently, further research is necessary to determine whether a true sampling rate above 10 Hz provides further improvement in the validity and reliability of GPS for measuring team sport-specific movement patterns [16].

Unlike GPS, where numerous validation studies exist [12], there are relatively few studies that have evaluated LPS for determining the movement patterns in team sports [1, 2, 4, 6, 8, 17]. Compared to GPS, LPS uses a locally deployed infrastructure [1], which offers the following advantages: (a) higher sampling rates [8], potentially increasing the validity and reliability for determining team sport-specific movement patterns [4]; (b) allowing measures not only indoors [8] but also in large stadia [6]; (c) more accurately detecting the actual player position [17]; (d) miniaturization of the devices, possibly also enabling ball tracking [18]; and based on both latter points (e) supporting tactical analyses [17]. However, due to different methodologies [6], it is difficult to compare the validity and reliability between the GPS and LPS from the literature. To date, there is only one study directly comparing the validity and reliability between both technologies for determining team sport-specific movement patterns [6]. Since the respective findings are impacted by some methodological limitations, including: (a) the use of low sample rate 4 Hz and 5 Hz GPS devices [12]; (b) data collection in a stadium in which the use of GPS is biased [19]; and (c) a lack of essential information concerning the GPS signal quality (i.e., horizontal dilution of precision) and data processing procedures (e.g., filtering techniques applied to the raw data) [16], more research comparing the validity and reliability between GPS and LPS for determining team sport-specific movement patterns is required [6].

Recently, a promising macroscopic biomechanical model for determining sprint mechanical properties during straight-line accelerated runs, based on an inverse dynamic approach applied to the center of mass, was proposed [20]. Using anthropometric and distance-time or velocity-time data, the theoretical maximal horizontal running velocity (V_max_), force (F_max_), and power output (P_max_) can be estimated [20]. Therefore, it is possible to compute linear force-velocity and parabolic power-velocity profiles that reflect the entire neuromuscular capacities to produce external force and power in a horizontal plane as a function of the underlying velocity [21]. For team sport athletes, these profiles may be useful to optimize their acceleration and sprint capacities via more individualized training drills [22]. While it has been shown in the original validation study (when compared to force plates) that timing gates allow a valid estimation of sprint mechanical properties during field conditions [20], one recent study reported poor validity for both 5 Hz and recently released 18 Hz GPS devices [23]. However, in this latter study, no timing gates but rather laser/radar derived velocities were used as the criterion measure [23].Therefore, and due to the potential superior data quality of LPS compared to GPS [17, 24], more research evaluating both technologies for estimating sprint mechanical properties during field conditions is warranted [23].

This study aimed to test the validity and reliability of GPS and LPS for measuring distances covered and sprint mechanical properties in team sports. Here, we evaluated two recently released 18 Hz GPS and 20 Hz LPS technologies together with one 10 Hz GPS technology for which the validity and reliability in team sports has been established [12]. The defined distances covered combined with the timing gates were used as the criterion measure: (a) to allow a comparison with numerous existing GPS/LPS validation studies, which used the same methodological approach [1, 6, 8, 12]; and (b) also because timing gates are the only available tool that has been directly validated against force plates to measure sprint mechanical properties during field conditions [20].

## Materials and methods

### Participants and ethics statement

Six male team sport athletes (age: 27±2 years, stature: 1.77±0.04 m, and body mass: 80.0±2.8 kg, 3–4 training sessions per week) took part in the study. The athletes were further characterized by the following performance data that were assessed as previously described [25]: body fat: 14.5±3.4%; maximum oxygen uptake: 48.8±4.7 ml/min/kg; and counter movement jump height: 38.9±3.9 cm. The athletes were informed of the purposes, procedures, and potential risks of the study and provided written consent of their approval to participate. All of the procedures were pre-approved by the Ethics Committee of the University of Wuppertal and were conducted in accordance with the Declaration of Helsinki.

### Experimental design

**Fig 1:**
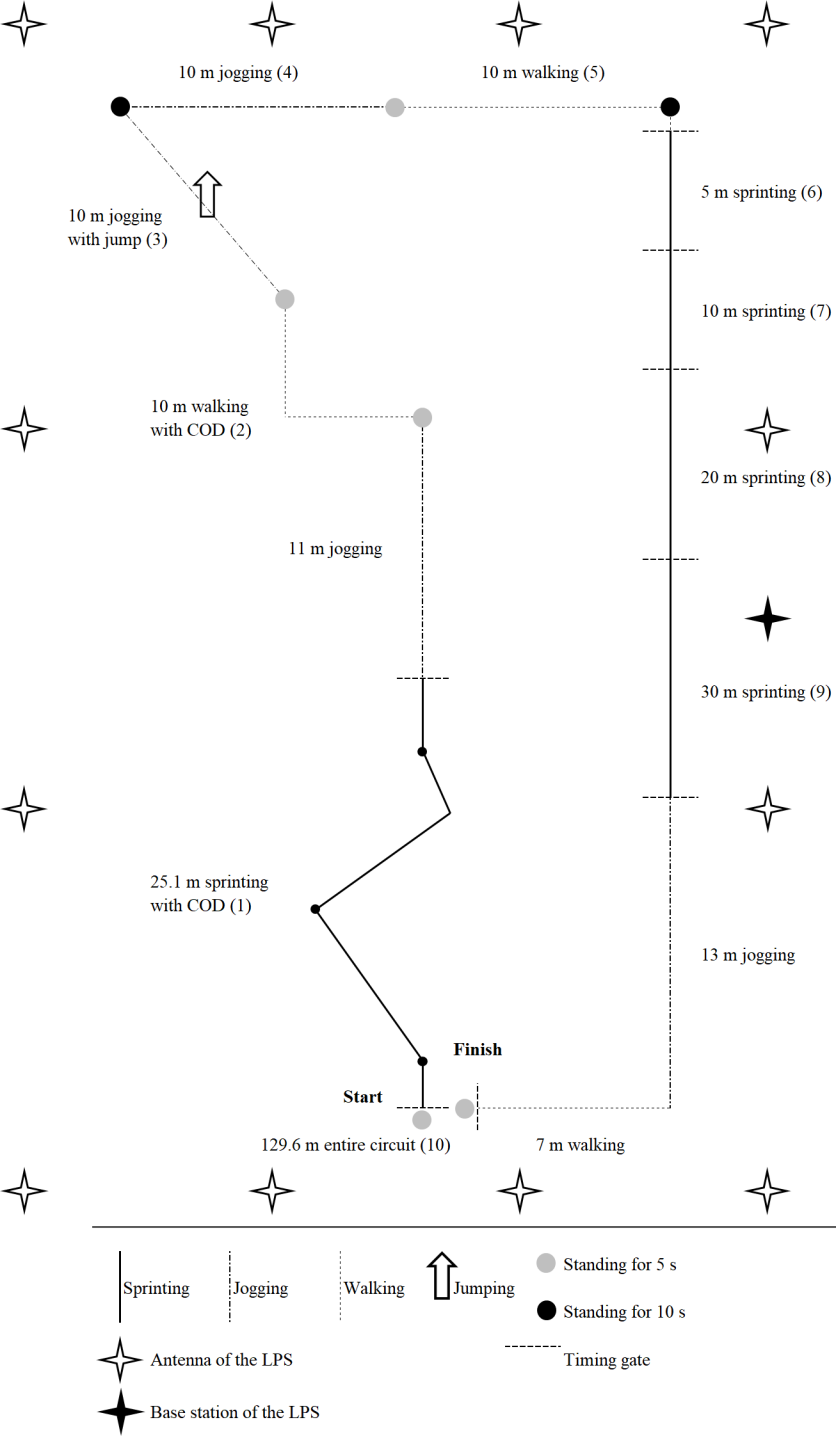
The design of the circuit and the setup of the LPS. LPS = Local positioning system. The particular sections that were used for determining the distances covered and sprint mechanical properties were numbered to allow a better assignment of the results provided in the tables and figures.

In line with previous studies [13, 26, 27], the validity and reliability of the positioning systems were examined by a circuit that was designed to mimic the fundamental movement patterns of most team sports in a standardized manner. The circuit consisted of various walking, jogging, and sprinting sections that were performed either in straight-lines or with CODs. Since movement patterns in many team sports also involve jumps [28, 29], a horizontal jump over 1 m and performed as high as possible was included in one jogging section to better understand the influence of jumps on GPS and LPS derived distance measures, which has been overlooked by previous studies. To simulate the intermittent nature of team sports and to facilitate the data processing, standing periods over 5 s and 10 s were also included. The design of the circuit is shown in Fig 1.

The circuit was conducted outdoors on a dry artificial-turf soccer field in suitable weather conditions (i.e., 20.8°C temperature, 41.4% humidity, and a completely clear sky). After self-paced warm-up procedures for 15 min and one familiarization trail, each athlete performed 10 repetitions of the circuit, which resulted in a total of 60 measures. For each repetition, the athletes were instructed: (a) to follow the marked path of the circuit as accurately as possible; (b) to maximally perform all of the sprints and jumps; (c) to reproduce the walking and jogging velocities; and (d) to stand still without upper body movements during the corresponding stationary periods. A 2 min passive recovery period separated each repetition. The duration of each standing and recovery period was acoustically controlled. Whilst the effort of the athletes was monitored by physiological measures during (i.e., via the heart rate) and after each repetition (i.e., via the capillary blood lactate concentration and rating of perceived exertion), using methods described elsewhere [30], the capacities-skills to reproduce the circuit was assessed via timing gate measures (i.e., via the time to complete the entire circuit and each separate sprinting section).

During the circuit, the athletes wore two devices of each positioning system between the scapula in a custom-made sport-shirt. Beneath the sport-shirt, they also wore a tight-fitting compression-shirt to minimize uncontrolled device movements [16]. To exclude noise induced by the electronic radiation of the devices [16], pilot tests showed that a between-device distance of at least 7 cm was required. Consequently, all of the devices were separated on the sport-shirt by 10 cm to eliminate this error source, which is a crucial technical aspect that has not been considered by previous GPS studies [16].

To evaluate the validity of the positioning systems, defined distances combined with timing gates were used as the criterion measures. Therefore, within the circuit, all of the distances that were performed in a straight-line were measured with a measuring tape. For the sprinting section with CODs (Fig 1, section 1), 10 repeated trundle wheel measures were used to correct the theoretical distance (i.e., 22 m) for the additional distance covered through the four CODs [25], which was 3.1±0.1 m. Thus, the distance of the sprinting section with CODs was corrected to 25.1 m. For the data processing, all of the sprinting sections (Fig 1, section 1 and 6–9) were equipped with double-light timing gates (Werthner Sport Consulting, TDS, Linz, Austria) positioned at 85 cm and 105 cm above the ground, respectively. For the sprinting sections, after standing still without upper body movements for 5 s, the athletes started 0.5 m before the first gate (Fig 1) as previously described [22].

To evaluate the reliability of the positioning systems, the differences between the two devices of each system were investigated (i.e., the between-device reliability) as previously performed in many GPS studies [12] but not investigated in any LPS study to date. To eliminate systematic biases grounded on the left or right positioning of the devices on the body, especially during the sprinting section with CODs, their positions were balanced through the entire data assessment (i.e., within the athletes).

### GPS and LPS technologies

To show potential progress, and in particular, to determine whether a true sampling rate above 10 Hz further improves the validity and reliability of the GPS [12], recently released 18 Hz devices (EXELIO srl, GPEXE PRO, version M03, Udine, Italy) [31] were evaluated together with established 10 Hz devices (Catapult Innovations, MinimaxX S4, version 6.71, Melbourne, Australia) [32]. While the exact sampling rate of the recently released GPS devices is 18.18 Hz, we rounded the sampling rate to 18 Hz for allowing a more fluid reading. The 10 Hz devices were chosen as a representative GPS standard because these devices are frequently used in team sport practice and applied studies [33] and in numerous previous validation studies [12], which collectively showed that these devices currently allow the most valid and reliable GPS assessment of team sport specific measures [12]. In accordance with previous studies [10, 34], all GPS devices were activated 15 min prior to the data collection to allow for satellite lock, and the signal quality was determined via both the number of connected satellites and horizontal dilution of precision [16]. Both GPS technologies measured the instantaneous velocity via the Doppler-shift (i.e., from the changes in the time signals emitted by the satellites) [16] as reported by the manufactures [31, 32].

To compare the validity and reliability between LPS and GPS technologies [6], one recently released LPS (KINEXON Precision Technologies, KINEXON ONE, version 1.0, Munich, Germany) [35] was selected. This LPS was chosen because it operates at 20 Hz and allows (from this technical aspect) a comparison with the latest GPS using a similar sampling rate. The LPS was installed, calibrated, and checked for its accuracy by one technician from the manufacturer. Four meters around the circuit, 12 antennae and one base station were positioned at four meters above the ground. The devices worn by the athletes transmitted time signals via radio-technology to the antennae, which sent the signals forward via a wide local area network (WLAN) to the base station. Using all of the signals, the base station then calculated the actual x,y position of the devices within the circuit [35]. Subsequently, instantaneous velocities were computed by positional differentiation (i.e., distance over time, whereas the distance was obtained from the changes in the x,y positions within each signal) [16]. According to previous LPS studies [4, 8] and for simulating the data traffic, for example, of two soccer teams, 20 devices randomly placed on the ground within the circuit were additionally activated during the data collection. The setup of the LPS is also shown in Fig 1.

### Data processing

All data processing procedures were applied to the reported velocity data and measured split times derived by the positioning systems and timing gates, respectively, using custom-made spreadsheets incorporating macro-based calculations (Microsoft, Excel 2016, Redmond, WA, USA).

First, all velocity data, reported by the respective proprietary softwares (10 Hz GPS: Catapult Innovations, Sprint, version 5.1.4, Melbourne, Australia; 18 Hz GPS: EXELIO srl, GPEXE Web Application, version 2.7.34, Udine, Italy; 20 Hz LPS: KINEXON Precision Technologies, Kinexon Web Application, version 3.2.6, Munich, Germany), were passed through a low-pass Butterworth digital filter to eliminate noise.The filter was applied with a 1 Hz cut-off frequency and two passes as previously applied in GPS [36] and LPS [4] studies. Due to the large impact of the filtering techniques and also due to the unknown filtering specifications of the manufacturers [16], all reported velocity data were consistently manually filtered to allow a fair comparison between the technologies [4] and replication by other researchers [16].

From the filtered velocity data and their integration over the time, the distances covered during each single section of interest within the circuit were computed. Therefore, a velocity threshold of 0.2 m/s was used to detect the start and end of the walking and jogging sections (Fig 1, section 2–5) as previously suggested [37, 38], whereas a threshold of 2.0 m/s was applied to determine the start of the sprinting sections (Fig 1, section 1 and 6–9). The 2.0 m/s threshold was chosen because our athletes covered 48±1 cm before reaching this threshold (as measured on average by all positioning systems), thus allowing us to correct for the 0.5 m distances covered from the beginning of the sprints until the first timing gate, which has not been considered by previous studies. The end of each sprinting section was obtained from the timing gate information.

**Fig 2:**
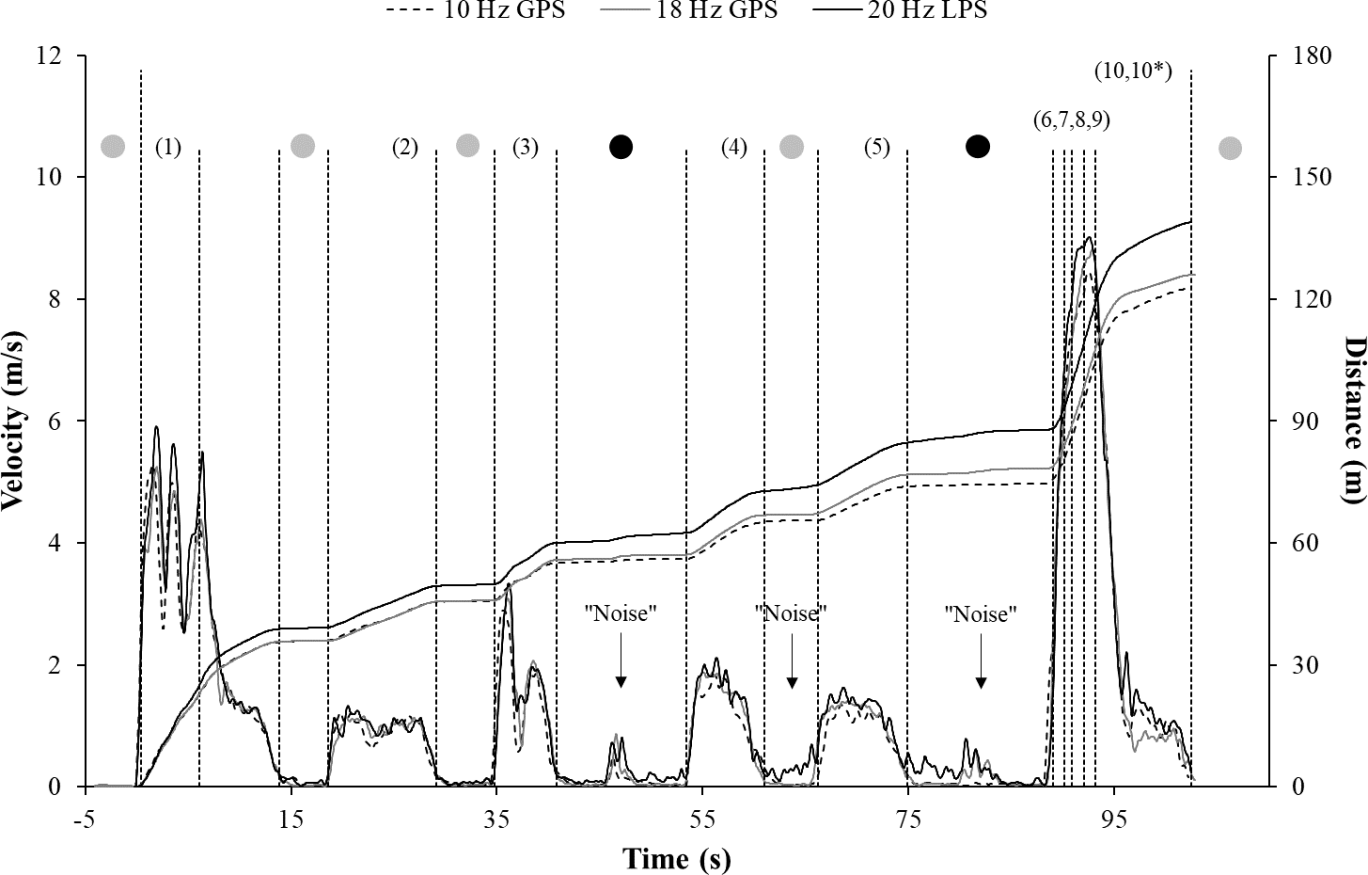
Exemplary plots of the filtered velocity data and resulting distances covered as measured by the positioning systems during the circuit. GPS = Global positioning system; LPS = local positioning system. While the number in brackets presents the section of the measurement within the circuit, the colored points show the standing phases(Fig 1). The vertical lines indicate the thresholds that were used to detect the start and end of the section within the circuit (see text). The entire distance covered was calculated twice: from the start to the end of the circuit (10) and through the summation of the single walking, jogging, and sprinting sections to correct for the noise observed during standing (10*).

However, visual inspection of the GPS and LPS derived velocity data showed that there was considerable noise during standing, which resulted in a false accumulation of the distance covered. Therefore, the entire distance covered was calculated via two approaches: (a) from the start until the end of the circuit using the velocity threshold of 0.2 m/s; and (b) through the summation of the distances covered during each single section involving walking, jogging, and sprinting (i.e., without the standing phases). Through the differences between these two approaches, it was possible to quantify the observed noise during standing, which has not been previously reported. Three exemplary plots of the filtered velocity data and the resulting distance covered measured by the positioning systems during the circuit are presented in Fig 2.

Finally, the sprint mechanical properties measured by the positioning systems and those assessed by the timing gates for comparisons were modeled as previously described in detail [20]. Therefore, the filtered velocity data and split times assessed during the sprinting section, which was performed in a straight-line (Fig 1, section 6–9), were analyzed. The following basic acceleration- (Eq 1), velocity- (Eq 2), and distance-time (Eq 3) functions were used [20]:

$$a(t)=\left(\frac{V_{max}}{\tau }\right)∙e^{-\frac{t}{\tau }}$$ (1)

$$v(t)=V_{max}∙(1-e^{-\frac{t}{\tau }})$$ (2)

$$x(t)=V_{max}∙\left(t-\tau +\tau ∙e^{-\frac{t}{\tau }}\right),$$ (3)

where V_max_ is the theoretical maximal horizontal running velocity reached at the end of the acceleration phase and τ is the acceleration time constant.

For the positioning systems, the filtered velocity data were fitted by the following function derived from Eq (2):

$$v(t)=V_{max}∙\left(1-e^{-\frac{t}{\tau }+\ln\left(1-\frac{v_{0}}{V_{max}}\right)}\right).$$ (4)

Then, the V_max_ and τ were determined by the least-square regression method. Since it is known that velocity data during the initial acceleration phase are less reliable [39], and in line with our velocity threshold to determine sprinting distances covered, only data with a velocity ≥2.0 m/s (i.e., v_0_ in Eq 4) were included in the fitting procedure. Importantly, the missing velocity data from 2 m/s to 0 m/s were interpolated. This procedure was necessary to consider for the unknown start times. For the timing gates and in consideration that our athletes covered 0.5 m before reaching the first gate (i.e., t_0_ in Eq 5), the measured split times were corrected to determine the unknown start times, and distances covered were fitted based on Eq (3) accordingly:

$$x(t+t_{0})=\frac{x_{0}}{\left(t_{0}-\tau +\tau ∙e^{-\frac{t_{0}}{\tau }}\right)}∙\left(t+t_{0}-\tau +\tau ∙e^{-\frac{t+t_{0}}{\tau }}\right).$$ (5)

Then, the t_0_ and τ were again determined by the least-square regression method using the known distances at 5.5 m, 10.5 m, 20.5 m, and 30.5 m and split times (i.e., t_1_-t_4_) as inputs. Finally, the V_max_ was defined as the mean of the velocity at t_0_-t_4_ using Eq (3).

For the positioning systems and timing gates, all of the velocity data were finally computed from 0 s to 5 s using Eq (2). Thereon, the theorectical horizontal force (F) over time was modeled as follows [20]:

$$F(t)=m∙a(t)+F_{aero}(t),$$ (6)

where m is the measured body mass and F_aero_ is the required aerodynamic drag force to overcome during sprinting. The instantaneous F_aero_ was calculated based on the measured environmental conditions during the data assessment,body height, and underlying running velocity as previously reported [20]. Then, the theoretical horizontal power output (P) at each instance was computed as follows [20]:

P(t)=F(t)∙v(t). (7)

**Fig 3:**
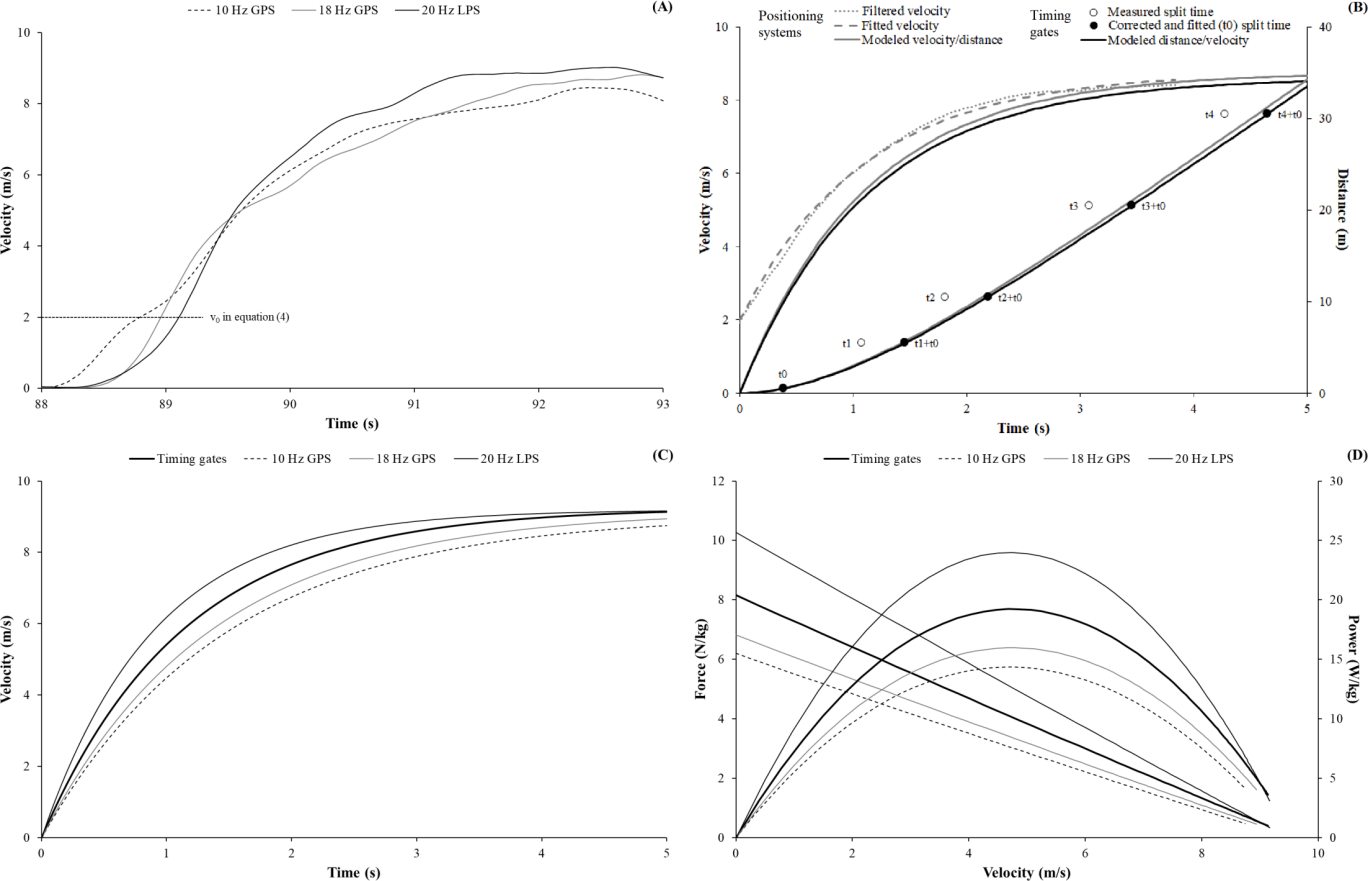
**Exemplary plots for our applied data processing to model the sprint mechanical properties via the filtered velocity data and split times measured by the positioning systems and timing gates, respectively (B). For each technology, an exemplary plot for the filtered velocity data (A), modeled velocity data (C), and derived sprint mechanical properties (D) is also shown.** GPS = Global positioning system; LPS = local positioning system. In (B), the grey functions show the data processing procedures applied to the filtered velocity data (A) measured by the positioning systems (i.e., exemplary shown for the LPS), whereas the black data represent the procedures applied to the split times assessed by the timing gates (see text). In (D), the theoretical horizontal force and power output is plotted over the modeled velocity (C) to exemplary show linear force-velocity and parabolic power-velocity profiles.

For statistical analyses, the theorectical maximal horizontal velocity (V_max_), force (F_max_), and power output (P_max_) were analyzed. The applied data processing for modelling the sprint mechanical properties from the filtered velocity data and split times is visually summarized in Fig 3. For each technology, an exemplary plot for the filtered velocity data, modeled velocity data, and derived sprint mechanical properties is also shown.

### Statistical analysis

For all statistical analyses, pre-built spreadsheets (Microsoft, Excel 2016, Redmond, WA, USA) for calculating measures of validity and reliability [40] as well as differences in means [41] were used.

First, outliers due to measurement errors (e.g., caused by empty batteries or poor signal strengths) were excluded from the data sets. Outliers were defined as those values that were ≥ the mean ± the two-fold pooled standard deviation as previously performed in a GPS validation study [10]. Then, the descriptive data were calculated as the mean and 90% confidence interval (CI) for each single device and also for each positioning system by combining the results from both devices. Validity calculations were performed for the two pooled devices in comparison with the criterion measure, whereas reliability computations required an investigation of the differences between both devices of each positioning system (i.e., the between-device reliability).

While differences were consistently analyzed to detect systematic biases, the typical error of estimate (TEE) and typical error (TE) were computed for validity and reliability purposes, respectively [7]. To allow a comparison of our data not only between but also within each positioning system, the biases were consistently calculated in relative terms, whereas the TEE and TE were determined in both absolute and relative units. The relative TE was calculated as a coefficient of the variation (CV) [7]. Presently, there is no consensus regarding statistical thresholds that would indicate acceptable validity and reliability in this field of research [12]. However, as suggested in a recent review summarizing the evidence of all of the available GPS evaluating studies, the relative measures of validity and reliability were rated as *good* (TEE or CV: 0 to <5%), *moderate* (TEE or CV: 5 to <10%), and *poor* (TEE or CV: ≥10%) [12]. The trial-to-trial reliability of the athletes to reproduce the movement patterns within the circuit was also expressed and rated by the magnitude of the CV.

To potentially increase the understanding of our calculated validity and reliability measures, the signal quality indices and noise levels between both GPS and all positioning systems, respectively, were also compared. The differences in the number of satellites, horizontal dilution of precision, and distances covered during standing were analyzed by magnitude based inferences as described in detail elsewhere [7]. Briefly, the dispositions of the 90% CIs for the mean differences in relation to the smallest worthwhile differences (i.e., the pooled standard deviations multiplied by 0.2) were analyzed. Then, the likelihoods for the mean of one positioning system being higher, similar, or lower than the mean of the other positioning system were determined and qualitatively described using the following probabilistic scale: <1%, *most unlikely*; 1 to <5%, *very unlikely*; 5 to <25%, *unlikely*; 25 to <75%, *possibly*; 75 to <95%, *likely*; 95 to <99%, *very likely*, and ≥99%, *most likely*. If the likelihoods for being both higher and lower were ≥5%, the differences were described as *unclear*. Otherwise, the differences were interpreted according to the observed likelihoods [7]. To clarify the meaningfulness of the differences, effect sizes according to Cohen’s d were calculated and interpreted accordingly: 0.0 to <0.2, *trivial*; 0.2 to <0.6, *small*; 0.6 to <1.2, *medium*; 1.2 to <2.0, *large*; 2.0 to <4.0, *very large*; and ≥4.0, *extremely large* [7].

## Results

### Physiological effort and trial-to-trial reliability

During the circuit, the mean heart rate of the athletes was 85.6±2.6% of their maximum. The mean capillary blood lactate concentration and rating of the perceived exertion was 10.2±2.9 mmol/l and 14.4±0.6, respectively.

Overall, there was a *good* trial-to-trial reliability of the athletes to reproduce the movement patterns within the circuit as determined by their times for completing the entire circuit and each separate sprinting section (Fig 1). The CVs of the times for completing the entire circuit over 129.6 m and sprinting over 25.1 m with CODs were 1.6±0.7% and 1.6±0.8%, respectively. The CVs of the times for sprinting over 5 m, 10 m, 20 m, and 30 m were 2.9±1.7%, 2.2±1.1%, 3.1±3.2%, and 1.6±0.8%, respectively.

### Number of outliers

For the 10 Hz GPS, 18 Hz GPS, and 20 Hz LPS, 12, 24, and 19 data files were classified as outliers, respectively. Therefore, and considering that 120 data files were collected in total for each positioning system, the relative loss of data sets due to measurement errors was 10.0%, 20.0%, and 15.8%, respectively.

### GPS signal quality

During the data collection and across both devices, the 10 Hz devices were connected to *most likely* a greater number of satellites (12.5±0.5) than the 18 Hz devices (9.6±0.5). The effect size of this difference was *extremely large* (d = 5.6). The difference in the horizontal dilution of precision between the two GPS technologies was *trivial* (10 Hz: 1.0±0.1; 18 Hz: 1.0±0.1; *small* effect size, d = 0.4). Between the two devices for each technology, all of the differences in regards to the number of satellites and horizontal dilution of precision were *trivial* along with *trivial* effect sizes (d≤0.1).

### Noise during standing

As quantified by the distances covered during standing (Fig 2), the 20 Hz LPS (6.9±0.5 m) had *most likely* more noise than either GPS technologies (18 Hz GPS: 3.9±0.7 m; 10 Hz GPS: 3.5±1.8 m) along with *very large* effect sizes (d = 2.4–3.6). Furthermore, the 18 Hz GPS had *very likely* more noise compared to the 10 Hz GPS; however, the effect size was *small* (d = 0.3). Relating to the entire distance covered over 129.6 m, the relative noises for the 10 Hz GPS, 18 Hz GPS, and 20 Hz LPS were 2.7±1.4%, 3.0±0.6%, and 5.3±0.7%, respectively.

### Descriptive data

Table 1 shows the descriptive data expressed as the means±90% CIs of the positioning systems for determining the distances covered and sprint mechanical properties. The data of the criterion measures are also presented.

**Table 1:** Descriptive data (mean±90% CI) of the positioning systems for determining the distances covered and sprint mechanical properties.

Criterion variables§ (section of measurement within the circuit; Fig 1)	10 Hz GPS			18 Hz GPS			20 Hz LPS		
	#1 (n = 49)	#2 (n = 49)	#1&2 (n = 108)	#1 (n = 43)	#2 (n = 43)	#1&2 (n = 96)	#1 (n = 46)	#2 (n = 46)	#1&2 (n = 101)
**25.1 m sprinting with CODs (1)**	22.0±0.3	22.1±0.2	22.0±0.2	22.6±0.1	22.8±0.1	22.7±0.1	24.6±0.1	24.5±0.1	24.6±0.1
**10 m walking with COD (2)**	9.3±0.2	9.6±0.1	9.5±0.1	9.8±0.0	9.8±0.1	9.8±0.0	10.6±0.1	10.6±0.1	10.6±0.0
**10 m jogging with jump (3)**	10.1±0.3	10.4±0.2	10.2±0.2	10.1±0.1	10.2±0.1	10.2±0.0	10.4±0.0	10.4±0.0	10.4±0.0
**10 m jogging (4)**	9.5±0.2	9.9±0.1	9.7±0.1	9.3±0.2	9.5±0.1	9.4±0.1	10.5±0.1	10.7±0.2	10.6±0.1
**10 m walking (5)**	9.4±0.2	9.5±0.1	9.5±0.1	9.7±0.1	9.9±0.1	9.8±0.0	10.8±0.2	10.9±0.1	10.8±0.1
**5 m sprinting (6)**	4.3±0.1	4.4±0.1	4.3±0.1	4.4±0.1	4.4±0.1	4.4±0.1	4.9±0.1	4.9±0.1	4.9±0.0
**10 m sprinting (7)**	8.9±0.2	8.7±0.2	8.8±0.1	8.9±0.1	9.0±0.1	8.9±0.1	10.1±0.1	10.1±0.1	10.1±0.0
**20 m sprinting (8)**	18.4±0.2	18.1±0.2	18.2±0.2	18.3±0.2	18.4±0.2	18.2±0.2	20.4±0.1	20.4±0.1	20.4±0.1
**30 m sprinting (9)**	28.1±0.2	27.8±0.3	27.9±0.2	28.0±0.3	28.1±0.2	27.9±0.2	30.4±0.1	30.4±0.1	30.5±0.1
**129.6 m entire circuit (10)**	125.7±0.9	128.3±0.6	126.9±0.6	127.0±0.4	128.4±0.4	127.6±0.3	138.5±0.6	139.6±0.4	139.0±0.3
129.6 m entire circuit (10*)	122.0±1.0	125.1±0.7	123.4±0.7	123.7±0.4	124.4±0.5	123.7±0.3	131.9±0.4	132.5±0.3	132.1±0.3
**1.1±0.0 s for τ (6–9)**	1.3±0.0	1.3±0.0	1.3±0.0	1.3±0.1	1.3±0.0	1.3±0.0	1.0±0.0	1.0±0.0	1.0±0.0
**8.2±0.1 m/s for V**_**max**_ **(6–9)**	8.3±0.1	8.2±0.1	8.2±0.1	8.2±0.2	8.2±0.1	8.2±0.1	8.4±0.1	8.5±0.1	8.4±0.1
**7.7±0.1 N/kg for F**_**max**_ **(6–9)**	6.4±0.3	6.2±0.2	6.3±0.2	6.2±0.2	6.3±0.2	6.2±0.1	8.9±0.2	8.9±0.2	8.9±0.1
**16.1±0.4 W/kg for P**_**max**_ **(6–9)**	13.6±0.8	12.9±0.5	13.2±0.4	13.0±0.5	13.2±0.6	13.0±0.4	19.1±0.7	19.0±0.5	18.9±0.4

GPS = Global positioning system; LPS = Local positioning system; #1 = Device 1; #2 = Device 2; CI = Confidence interval; COD = Change of direction; τ = Acceleration time constant; V_max_ = Theoretical maximal running velocity; F_max_ = Theoretical maximal horizontal force; P_max_ = Theoretical maximal horizontal power output.

§ = The criterion data for the distances covered and sprint mechanical properties are reported as the true distances and overall means±90% CIs across all trials, respectively.

* = Entire distance covered calculated through the summation of the single sections of the circuit (i.e., without the noise during standing; Fig 2).

### Validity for distances covered and sprint mechanical properties

Table 2 summarizes the calculated relative biases and absolute TEEs that were computed to quantify the validity of the positioning systems for determining the distances covered and sprint mechanical properties. Excluding jogging over 10 m with a jump and τ, there were negative biases of both GPS technologies, whereas positive biases was found for the LPS with the exception of sprinting over 25.1 m with CODs, sprinting over 5 m, and τ. Overall, for the 10 Hz GPS, there were larger biases and TEEs followed by the 18 Hz GPS and 20 Hz LPS. These findings were most evident for the entire distance covered over 129.6 m, sprinting over 25.1 m with CODs, and sprinting over 5–30 m as well as theoretical F_max_ and P_max_ in consideration of only the TEEs.

**Table 2:** Validity (relative biases and absolute TEEs) of the positioning systems for determining the distances covered and sprint mechanical properties.

Criterion variables§ (section of measurement within the circuit; Fig 1)	10 Hz GPS		18 Hz GPS		20 Hz LPS	
	#1&2 (n = 108)		#1&2 (n = 96)		#1&2 (n = 101)	
	Bias (%)±90% CI	TEE (Unit)±90% CI	Bias (%)±90% CI	TEE (Unit)±90% CI	Bias (%)±90% CI	TEE (Unit)±90% CI
**25.1 m sprinting with CODs (1)**	-11.7±0.7	1.0±0.1	-9.2±0.3	0.5±0.1	-2.1±0.3	0.4±0.1
**10 m walking with COD (2)**	-5.0±1.2	0.7±0.1	-1.9±0.3	0.2±0.0	+6.1±0.4	0.2±0.0
**10 m jogging with jump (3)**	+1.9±1.7	1.0±0.1	+1.8±0.4	0.3±0.0	+4.2±0.3	0.2±0.0
**10 m jogging (4)**	-3.3±1.2	0.7±0.1	-6.1±1.0	0.5±0.1	+5.7±1.0	0.6±0.1
**10 m walking (5)**	-5.5±1.0	0.6±0.1	-2.2±0.5	0.3±0.0	+8.4±1.0	0.6±0.1
**5 m sprinting (6)**	-13.0±1.6	0.5±0.1	-11.8±1.1	0.3±0.0	-1.6±0.7	0.2±0.0
**10 m sprinting (7)**	-11.9±1.3	0.8±0.1	-10.6±0.9	0.5±0.1	+1.3±0.5	0.3±0.0
**20 m sprinting (8)**	-8.9±0.9	1.1±0.1	-8.8±1.0	1.0±0.1	+2.0±0.3	0.4±0.0
**30 m sprinting (9)**	-6.8±0.6	1.1±0.1	-6.7±0.6	1.0±0.1	+1.5±0.2	0.3±0.0
**129.6 m entire circuit (10)**	-2.1±0.4	3.6±0.4	-1.6±0.3	1.9±0.2	+6.2±0.2	1.9±0.2
129.6 m entire circuit (10*)	-4.7±0.5	4.2±0.5	-4.5±0.3	1.9±0.2	+1.9±0.2	1.6±0.2
**1.1±0.0 s for τ (6–9)**	+24.7±4.1	0.2±0.0	+25.4±3.1	0.2±0.0	-10.0±1.5	0.1±0.0
**8.2±0.1 m/s for V**_**max**_ **(6–9)**	0.0±0.6	0.3±0.0	-0.2±0.7	0.4±0.0	+2.1±0.3	0.2±0.0
**7.7±0.1 N/kg for F**_**max**_ **(6–9)**	-16.6±2.5	1.3±0.2	-17.0±1.7	0.9±0.1	+15.1±1.6	0.7±0.1
**16.1±0.4 W/kg for P**_**max**_ **(6–9)**	-16.3±2.3	2.5±0.3	-16.9±1.5	1.5±0.2	+17.7±1.3	1.3±0.1

GPS = Global positioning system; LPS = Local positioning system; #1 = Device 1; #2 = Device 2; TEE = Typical error of estimate; CI = Confidence interval; COD = Change of direction; τ = Acceleration time constant; V_max_ = Theoretical maximal running velocity; F_max_ = Theoretical maximal horizontal force; P_max_ = Theoretical maximal horizontal power output.

§ = The criterion data for the distances covered and sprint mechanical properties are reported as the true distances and overall means±90% CIs across all trials, respectively.

* = Entire distance covered calculated through the summation of the single sections of the circuit (i.e., without the noise during standing; Fig 2).

**Fig 4:**
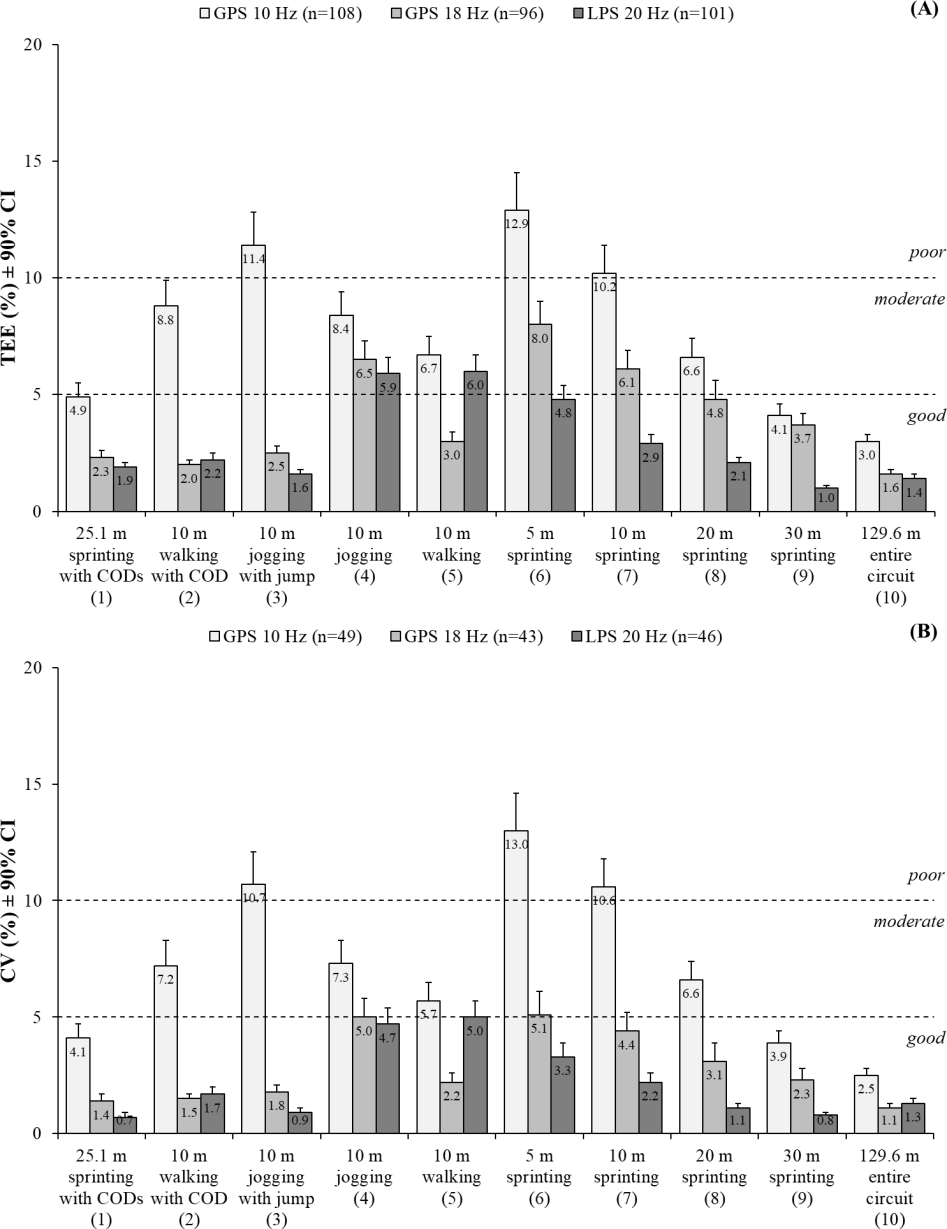
**Validity (relative TEEs; A) and reliability (relative TEs presented as CVs; B) of the positioning systems for determining the distances covered.** GPS = Global positioning system; LPS = local positioning system; TEE = Typical error of estimate; TE = Typical error; CV = Coefficient of variation; CI = Confidence interval; COD = Change of direction. The numbers in the brackets present the section of measurement within the circuit (Fig 1).

**Fig 5:**
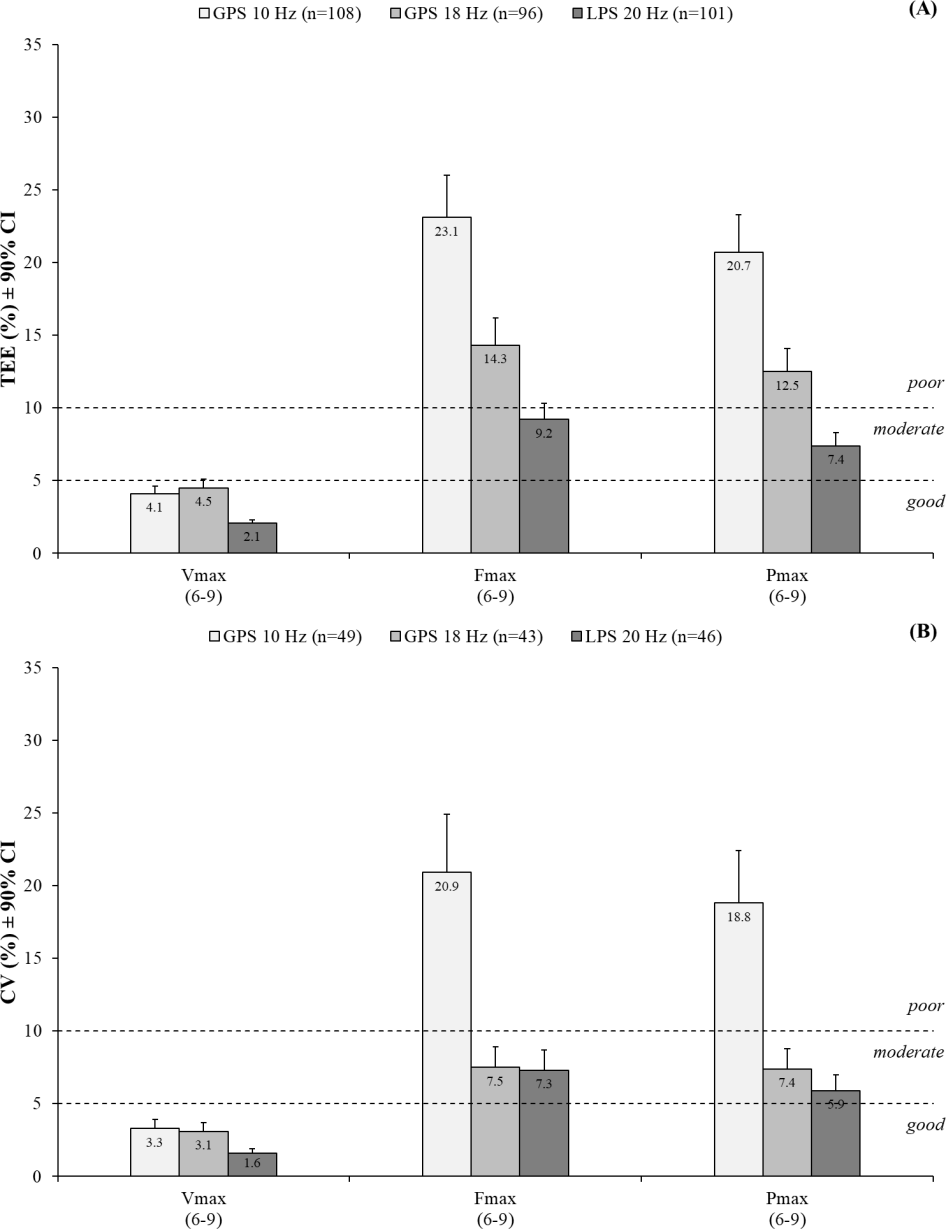
**Validity (relative TEEs; A) and reliability (relative TEs presented as CVs; B) of the positioning systems for determining the sprint mechanical properties.** GPS = Global positioning system; LPS = local positioning system; TEE = Typical error of estimate; TE = Typical error; CV = Coefficient of variation; CI = Confidence interval; V_max_ = Theoretical maximal running velocity; F_max_ = Theoretical maximal horizontal force; P_max_ = Theoretical maximal horizontal power. The numbers in the brackets present the section of measurement within the circuit (Fig 1).

Figs 4A and 5A show the relative TEEs that were used to qualitatively rate the validity of the positioning systems. All positioning systems showed *good* validity for the entire distance covered over 129.6 m, sprinting over 25.1 m with CODs, and sprinting over 30 m. The 20 Hz LPS demonstrated *good* validity for sprinting over 5–20 m. Conversely, the 18 Hz GPS showed *moderate* validity for sprinting over 5–10 m and *good* validity over 20 m, whereas the 10 Hz GPS demonstrated *poor* and *moderate* validity, respectively. The 18 Hz GPS and 20 Hz LPS showed *good* validity for walking over 10 m with a COD and jogging over 10 m with a jump, whereas the 10 Hz GPS showed *moderate* and *poor* validity, respectively. All positioning systems demonstrated a *moderate* validity for jogging and walking over 10 m each with the exception of the 18 Hz GPS that showed *good* validity for the latter (Fig 4A).

All positioning systems demonstrated *good* validity for theoretical V_max_. While the 20 Hz LPS demonstrated *moderate* validity for theoretical F_max_ and P_max_, both GPS technologies showed *poor* validity for these measures (Fig 5A).

### Reliability for distances covered and sprint mechanical properties

Table 3 summarizes all calculated relative biases and absolute TEs that were computed to express the reliability of the positioning systems for determining the distances covered and sprint mechanical properties. Collectively, the 10 Hz GPS showed larger biases and TEs than the 18 Hz GPS and 20 Hz LPS. Again, these results were most clear for the entire distance covered over 129.6 m, sprinting over 25.1 m with CODs, sprinting over 5–30 m as well as theoretical F_max_ and P_max_.

**Table 3:** Reliability (relative biases and absolute TEs) of the positioning systems for determining the distances covered and sprint mechanical properties.

Criterion variables§ (section of measurement within the circuit; Fig 1)	10 Hz GPS		18 Hz GPS		20 Hz LPS	
	#1vs.2 (n = 49)		#1vs.2 (n = 43)		#1vs.2 (n = 46)	
	Bias (%)±90% CI	TE (Unit)±90% CI	Bias (%)±90% CI	TE (Unit)±90% CI	Bias (%)±90% CI	TE (Unit)±90% CI
**25.1 m sprinting with CODs (1)**	-0.5±1.3	0.9±0.1	-0.8±0.5	0.3±0.1	+0.1±0.2	0.2±0.0
**10 m walking with COD (2)**	-3.0±2.1	0.6±0.1	-0.3±0.5	0.1±0.0	-0.3±0.6	0.2±0.0
**10 m jogging with jump (3)**	-2.1±3.2	1.0±0.2	-0.2±0.6	0.2±0.0	-0.1±0.3	0.1±0.0
**10 m jogging (4)**	-4.1±2.2	0.7±0.1	-2.6±1.6	0.4±0.1	-1.6±1.5	0.5±0.1
**10 m walking (5)**	-1.4±1.8	0.5±0.1	-1.6±0.8	0.2±0.0	-0.8±1.7	0.5±0.1
**5 m sprinting (6)**	-1.1±4.1	0.5±0.1	-1.2±1.8	0.2±0.0	+0.4±1.1	0.2±0.0
**10 m sprinting (7)**	+2.5±3.5	0.9±0.2	-0.6±1.6	0.4±0.1	+0.1±0.7	0.2±0.0
**20 m sprinting (8)**	+2.2±2.2	1.2±0.2	-0.2±1.1	0.5±0.1	0.0±0.4	0.2±0.0
**30 m sprinting (9)**	+1.2±1.3	1.1±0.2	-0.7±0.8	0.6±0.1	0.0±0.3	0.2±0.0
**129.6 m entire circuit (10)**	-2.0±0.8	3.0±0.5	-1.1±0.4	1.4±0.3	-0.8±0.4	1.7±0.3
129.6 m entire circuit (10*)	-2.4±0.9	3.4±0.6	-0.9±0.4	1.5±0.3	-0.4±0.4	1.4±0.2
**1.1±0.0 s for τ (6–9)**	+2.9±7.2	0.2±0.0	+2.7±3.1	0.1±0.0	-0.2±2.8	0.1±0.0
**8.2±0.1 m/s for V**_**max**_ **(6–9)**	+1.4±1.1	0.3±0.0	+0.6±1.1	0.3±0.0	-0.4±0.5	0.1±0.0
**7.7±0.1 N/kg for F**_**max**_ **(6–9)**	+6.3±6.8	1.3±0.2	-1.0±2.5	0.5±0.1	+1.0±2.5	0.6±0.1
**16.1±0.4 W/kg for P**_**max**_ **(6–9)**	+7.0±6.1	2.5±0.4	-0.4±2.6	1.0±0.2	+0.3±2.0	1.1±0.2

GPS = Global positioning system; LPS = Local positioning system; #1 = Device 1; #2 = Device 2; TE = Typical error; CI = Confidence interval; COD = Change of direction; τ = Acceleration time constant; V_max_ = Theoretical maximal running velocity; F_max_ = Theoretical maximal horizontal force; P_max_ = Theoretical maximal horizontal power output.

§ = The criterion data for the distances covered and sprint mechanical properties are reported as the true distances and overall means±90% CIs across all trials, respectively.

* = Entire distance covered calculated through the summation of the single sections of the circuit (i.e., without the noise during standing; Fig 2).

Figs 4B and 5B show the CVs that were calculated to qualitatively rate the reliability of the positioning systems. All positioning systems showed *good* reliability for the entire distance covered over 129.6 m, sprinting over 25.1 m with CODs, and sprinting over 30 m. The 20 Hz LPS showed *good* reliability for sprinting over 5–20 m. While the reliability of the 18 Hz GPS was *moderate* for sprinting over 5 m and *good* over 10–20 m, the reliability of the 10 Hz GPS was *poor* over 5–10 m and *moderate* over 20 m. The 18 Hz GPS and 20 Hz LPS showed a *good* reliability for walking over 10 m with a COD, jogging over 10 m with a jump, and jogging and walking over 10 m each. The reliability of the 10 Hz GPS was *poor* for jogging over 10 m with a jump as well as *moderate* for walking over 10 m with a COD and jogging and walking over 10 m each (Fig 4A).

All positioning systems demonstrated *good* reliability for theoretical V_max_. The 18 Hz GPS and 20 Hz LPS showed *good* reliability for theorectical F_max_ and P_max_, whereas the 10 Hz GPS demonstrated *poor* reliability for both measures (Fig 5B).

## Discussion

In response to the circuit, the mean heart rates, lactate concentrations, and ratings of perceived exertion of our athletes were comparable to those observed during match play in various team sports such as soccer [42], handball [29], rugby [43], basketball [44], and field hockey [45]. Since the CVs of the times for completing the entire circuit and each single sprinting section were all <5.0%, it can be concluded that our athletes showed high efforts [46] and were able to reproduce the movement patterns involved in the circuit [7]. Overall, this indicates that our circuit was appropriately designed to simulate fundamental movement patterns of team sports in a standardized manner, which is important to allow external valid conclusions for the positioning systems evaluated here.

Although different methodological approaches were used, our validity and reliability measures of the 10 Hz GPS and 20 Hz LPS for determining distances covered were comparable to those reported in previous GPS [11, 13, 47] and LPS studies [1, 8]. However, our statistical outcomes of the 18 Hz GPS for determining sprint mechanical properties were worse than those reported in one existing study, whereas the mutual relations of our validity and reliability measures for the theoretical V_max_, F_max_, and P_max_ were similar [23]. No further data can be compared with the literature.

In relation to the older video-camera or recent LPS, the GPS presently allows the most time-efficient measurements of movement patterns in team sports [6]. Considering the reliability and validity for determining team sport-specific movement patterns, previous research has shown that 5 Hz devices were superior than 1 Hz devices [27, 34] and 10 Hz devices were superior than 5 Hz devices [11, 48], whereas 15 Hz devices were unexpectedly not superior to 10 Hz devices [10, 13]. These observations suggest that the validity and reliability of the GPS is only affected by the sampling rate up to 10 Hz [12]. However, our study demonstrates that 18 Hz devices show an enhanced validity and reliability for determining distances covered and sprint mechanical properties compared to 10 Hz devices. Our findings were most evident for crucial team sport specific measures, such as the entire distance covered and short sprinting distances in a straight-line or with CODs, and also for new inverse dynamic modelled indices as theoretical F_max_ and P_max_ (Tables 2 and 3; Figs 4 and 5). The most reasonable explanation for these discrepancies is that previous studies have examined 15 Hz devices [10, 13–15] that actually up-sample a 5 Hz signal via linear interpolation [14], whereas our investigated devices operate at a true 18 Hz sampling rate, which potentially increase the chance to capture all of the relevant data during team sport-specific movement patterns, particularly changes in velocities [49]. Overall, a true sampling rate above 10 Hz further improved the validity and reliability of the GPS for determining movement patterns in team sports. Noteworthy, in addition to the sampling rate, other technical aspects may also influence the validity and reliability, including the antenna, microprocessor and its data processing algorithms, positioning of the devices on the body, and environmental conditions. Further research is warranted to investigate these factors [16].

Regarding environmental conditions potentially affecting the validity and reliability of the GPS [12, 16], it is commonly reported in applied [36, 50] and validation studies [10, 34] for two indices of the signal quality, namely, the number of connected satellites and horizontal dilution of precision. Without scientific evidence for these two indices, values ≥6 and ≤1, respectively, are considered ideal [16]. While our indices satisfy these criteria, it is worth mentioning that the more valid and reliable 18 Hz devices were connected to fewer satellites than the 10 Hz devices despite no difference in the horizontal dilution of precision. Overall, these remarks underline the need to develop separate signal quality thresholds for all of the available GPS devices, below/above which data should not be interpreted [16].

In contrast to the GPS that uses time signals emitted by satellites orbiting the earth [51], the LPS functions in the opposite manner [24] such that the devices send time signals to locally placed antennae [2]. This different measurement principle primarily allows the LPS to: (a) operate at higher sampling rates [4], which potentially enhances the validity and reliability for team sport specific measures [4]; (b) work outdoors and indoors in any field surrounded by the antennae [6]; and (c) miniaturize the devices (i.e., 49×33×8 mm and 15 g vs. 75×60×22 mm and 75 g vs. 88×50×19 mm and 88 g for the 20 Hz LPS [35], 18 Hz GPS [31], and 10 Hz GPS [32], respectively), mainly because there is no need for storing data within the devices [24]. In team sports, this miniaturization aspect is a key prerequisite when aiming to investigate official matches for performance analysis without disturbing the athletes, for example, as required by the FIFA [52], or to track the ball for tactical reasons [17] and combined with further objects, such as tibia guards, poles, or goals, also for technical skill analyses in the future [18].

The validity and reliability of the GPS and LPS for determining movement patterns in team sports has, to our knowledge, only been compared in one previous study, showing inconsistent findings for measuring distances covered and maximal acceleration and velocity data [6]. Additionally, this previous study compared a low sample rate of 4 Hz and 5 Hz GPS with a 45 Hz LPS [6], but unfortunately, this study resulted in no conclusion, which may indicate that the differences are related to clearly diverse sampling rates or measurement principles. With this in mind, a methodological strength of our study is that we compared one GPS and LPS technology operating at comparable sampling rates. Our outcomes demonstrate that the 20 Hz LPS had a superior validity and reliability for determining the distances covered and sprint mechanical properties than the 18 Hz GPS, and plausibly, also compared to the 10 Hz GPS. Again, the enhanced validity and reliability of the LPS was clearest for crucial team sport-specific measures (Tables 2 and 3; Figs 4 and 5). Our validity and reliability measures of the 20 Hz LPS for determining the distances covered were comparable with the those reported in previous studies investigating LPS technologies operating at obviously lower (i.e., 10 Hz) [1] and higher sampling rates (i.e., 45 Hz) [8]. Together, it can be expected that the improved validity and reliability of the LPS compared to the GPS is due to the different measurement principle [2], which potentially permits the assessment of positional and derived velocity data at a higher quality [24]. However, compared to the GPS, it is also important to mention that the LPS has two main limitations, especially for practical purposes including: (a) higher acquisition costs [4], which are mainly due to the more complex technical background; and (b) less flexibility [4] because it takes approximately 1–2 hours to install the antennae and calibrate the system. Additionally, it is noteworthy that the 20 Hz LPS, and also the 18 Hz GPS, had more outliers due to measurement errors compared to the 10 Hz GPS during our standardized conditions, which limits their practical applications at this time.

Our study also shows that the LPS had more noise than either of the GPS technologies as determined by the distances covered during standing (Fig 2), which can be seen as a further limitation. In our study, the observed noise can be characterized by two phenomena, namely, (a) a shift in the zero-velocity line; and (b) an increase in the velocity due to performed turning maneuvers (Fig 2). Since the second phenomenon was only evident in the 20 Hz LPS and 18 Hz GPS (Fig 2), it can be speculated that the 10 Hz GPS devices use their integrated inertial sensors to eliminate this noise source. However, concerning the first phenomenon, the shift in the zero-velocity line was solely present in the LPS (Fig 2). In this context, previous research suggests that the weather (e.g., heavy rain, fog, or snow) and environmental conditions (e.g., tall buildings or construction materials like metal) [2], filtering techniques (e.g., cut-off frequencies or number of passes) [4], and positioning of the devices in relation to the antennae (e.g., next to the antennae or in the middle of the field) [1] can impact LPS measurements. In our study, the weather and environmental conditions and applied filtering techniques were identical for all of the positioning systems, and therefore, the positioning of the LPS devices in relation to the antennae may explain why there was a shift in the velocity line. In fact, the shift was only observed during the standing periods separating both walking and jogging sections over 10 m (Fig 2, section 4 and 5), which were located at the corners of the circuit and close to the antennae (Fig 1). This close proximity combined with the possibility that the sent signals were partially blocked due to the positioning of the devices on the back of the athletes may result in an inferior geometric dilution of precision [1]. Thus, for the LPS, a promising approach to cope with in this particular noise source may be to apply position-specific filtering techniques incorporating the positions and distances of the devices in relation to the antennae, which should be evaluated in future studies.

In addition to running, movement patterns of many team sports also involve frequent jumps [28, 29]. However, previous research has failed to examine the influence of jumps on GPS and LPS derived distance measures. Our study shows that a jump clearly decreases the validity and reliability of the 10 Hz GPS for determining the distances covered compared to the 18 Hz GPS and 20 Hz LPS (Tables 2 and 3; Figs 4 and 5). Since it is known that 10 Hz GPS devices have limitations for measuring maximal changes in velocities [53], it can be speculated that the rapid changes in velocities occurring during the jump (Fig 2, section 3) within our circuit (Fig 1, section 3) account for these outcomes. These assumptions are important when aiming to investigate team sports involving numerous jumps, such as soccer [28], via our tested 10 Hz GPS. Overall, these notes suggest that further research for determining jumps via positioning systems in team sports is warranted, most promising through inertial sensors [54].

Previous research showed that the validity and reliability of GPS [12] and LPS [4, 8] decreased with higher movement velocities. Conversely, our statistical indices for walking and jogging over 10 m (Fig 2, section 4 and 5) were higher compared to those for sprinting over 25.1 m with CODs and sprinting over 30 m (Tables 2 and 3; Figs 4 and 5). Thereby, it is likely that the noise of the LPS had an impact (Fig 2), whereas both GPS technologies could have been influenced by few trees positioned approximately 10 m apart on the corresponding side of the circuit. However, trees next to one side of a soccer field can be seen as externally valid conditions for evaluating positioning systems in team sports.

While our study clearly increases the understanding regarding the validity and reliability of GPS and LPS technologies for determining movement patterns in team sports, it is worth mentioning that our findings were limited by the circumstance that no elite athletes took part. Therefore, the impact of higher velocity and acceleration/deceleration data, particularly reached by elite athletes, on our validity and reliability measures remains unknown. Since each athlete performed 10 trials of our circuit, there is also a possibility of a pseudo replication, which could have had an effect on our outcomes. More research to clarify these two aspects is needed.

## Conclusions

This study shows that 18 Hz GPS devices had better validity and reliability for determining distances covered and sprint mechanical properties than 10 Hz GPS devices. Additionally, compared with both GPS technologies, 20 Hz LPS technology had superior validity and reliability overall. However, compared to 10 Hz GPS, 18 Hz GPS and 20 Hz LPS technologies had more outlieres due to measurement errors, which limits their practical applications at this time. While differences between both GPS technologies are likely caused by different sampling rates, differences between the GPS and LPS technologies may be related to the different measurement principles. Importantly, for team sports, each positioning system has its advantages and disadvantages that should be considered regarding the specific objectives. However, since physical performance differences (e.g., between or within teams) and training effects (e.g., according to the pre- or in-season) are small on an elite team sport level, the most valid and reliable accessible positioning system, namely here, the 20 Hz LPS is recommended to use for meaningful decisions.

## Supporting information

S1 File Raw dataset. (XLSX)

## References

[1] Sathyan T, Shuttleworth R, Hedley M, Davids K. Validity and reliability of a radio positioning system for tracking athletes in indoor and outdoor team sports. Behav Res Methods. 2012;44(4):1108–14. doi: 10.3758/s13428-012-0192-2 22477436 10.3758/s13428-012-0192-2

[2] Siegle M, Stevens T, Lames M. Design of an accuracy study for position detection in football. J Sports Sci. 2013;31(2):166–72. doi: 10.1080/02640414.2012.723131 22994162 10.1080/02640414.2012.723131

[3] Aughey RJ. Applications of GPS technologies to field sports. Int J Sports Physiol Perform. 2011;6(3):295–310. 21911856 10.1123/ijspp.6.3.295

[4] Stevens TG, de Ruiter CJ, van Niel C, van de Rhee R, Beek PJ, Savelsbergh GJ. Measuring acceleration and deceleration in soccer-specific movements using a local position measurement (LPM) system. Int J Sports Physiol Perform. 2014;9(3):446–56. doi: 10.1123/ijspp.2013-0340 24509777 10.1123/ijspp.2013-0340

[5] Carling C. Interpreting physical performance in professional soccer match-play: should we be more pragmatic in our approach? Sports Med. 2013;43(8):655–63. doi: 10.1007/s40279-013-0055-8 23661303 10.1007/s40279-013-0055-8

[6] Buchheit M, Allen A, Poon TK, Modonutti M, Gregson W, Di Salvo V. Integrating different tracking systems in football: multiple camera semi-automatic system, local position measurement and GPS technologies. J Sports Sci. 2014;32(20):1844–57. doi: 10.1080/02640414.2014.942687 25093242 10.1080/02640414.2014.942687

[7] Hopkins WG, Marshall SW, Batterham AM, Hanin J. Progressive statistics for studies in sports medicine and exercise science. Med Sci Sports Exerc. 2009;41(1):3–13. doi: 10.1249/MSS.0b013e31818cb278 19092709 10.1249/MSS.0b013e31818cb278

[8] Frencken WG, Lemmink KA, Delleman NJ. Soccer-specific accuracy and validity of the local position measurement (LPM) system. J Sci Med Sport. 2010;13(6):641–5. doi: 10.1016/j.jsams.2010.04.003 20594910 10.1016/j.jsams.2010.04.003

[9] Gray AJ, Jenkins D, Andrews MH, Taaffe DR, Glover ML. Validity and reliability of GPS for measuring distance travelled in field-based team sports. J Sports Sci. 2010;28(12):1319–25. doi: 10.1080/02640414.2010.504783 20859825 10.1080/02640414.2010.504783

[10] Vickery WM, Dascombe BJ, Baker JD, Higham DG, Spratford WA, Duffield R. Accuracy and reliability of GPS devices for measurement of sports-specific movement patterns related to cricket, tennis, and field-based team sports. J Strength Cond Res. 2014;28(6):1697–705. doi: 10.1519/JSC.0000000000000285 24149747 10.1519/JSC.0000000000000285

[11] Rampinini E, Alberti G, Fiorenza M, Riggio M, Sassi R, Borges TO, et al Accuracy of GPS devices for measuring high-intensity running in field-based team sports. Int J Sports Med. 2015;36(1):49–53. doi: 10.1055/s-0034-1385866 25254901 10.1055/s-0034-1385866

[12] Scott MT, Scott TJ, Kelly VG. The Validity and Reliability of Global Positioning Systems in Team Sport: A Brief Review. J Strength Cond Res. 2016;30(5):1470–90. doi: 10.1519/JSC.0000000000001221 26439776 10.1519/JSC.0000000000001221

[13] Johnston RJ, Watsford ML, Kelly SJ, Pine MJ, Spurrs RW. Validity and interunit reliability of 10 Hz and 15 Hz GPS units for assessing athlete movement demands. J Strength Cond Res. 2014;28(6):1649–55. doi: 10.1519/JSC.0000000000000323 24276300 10.1519/JSC.0000000000000323

[14] Rawstorn JC, Maddison R, Ali A, Foskett A, Gant N. Rapid directional change degrades GPS distance measurement validity during intermittent intensity running. PLoS One. 2014;9(4):e93693 doi: 10.1371/journal.pone.0093693 24733158 10.1371/journal.pone.0093693 PMC3986049

[15] Buchheit M, Al Haddad H, Simpson BM, Palazzi D, Bourdon PC, Di Salvo V, et al Monitoring accelerations with GPS in football: time to slow down? Int J Sports Physiol Perform. 2014;9(3):442–5. doi: 10.1123/ijspp.2013-0187 23916989 10.1123/ijspp.2013-0187

[16] Malone JJ, Lovell R, Varley MC, Coutts AJ. Unpacking the black box: Applications and considerations for using GPS devices in sport. Int J Sports Physiol Perform. 2017;12(2):218–26. doi: 10.1123/ijspp.2016-0030 27736244 10.1123/ijspp.2016-0236

[17] Ogris G, Leser R, Horsak B, Kornfeind P, Heller M, Baca A. Accuracy of the LPM tracking system considering dynamic position changes. J Sports Sci. 2012;30(14):1503–11. doi: 10.1080/02640414.2012.712712 22906154 10.1080/02640414.2012.712712

[18] Seidl T, Czyz T, Spandler D, Franke N, Lochmann M. Validation of football's velocity provided by a radio-based tracking system. Procedia Engineering. 2016;147:584–9.

[19] Williams M, Morgan S. Horizontal positioning error derived from stationary GPS units: A function of time and proximity to building infrastructure. Int J Perform Analysis Sport. 2009;9(2):275–80.

[20] Samozino P, Rabita G, Dorel S, Slawinski J, Peyrot N, Saez de Villarreal E, et al A simple method for measuring power, force, velocity properties, and mechanical effectiveness in sprint running. Scand J Med Sci Sports. 2016;26(6):648–58. 25996964 10.1111/sms.12490

[21] Morin JB, Samozino P. Interpreting power-force-velocity profiles for individualized and specific training. Int J Sports Physiol Perform. 2016;11(2):267–72. doi: 10.1123/ijspp.2015-0638 26694658 10.1123/ijspp.2015-0638

[22] Buchheit M, Samozino P, Glynn JA, Michael BS, Al Haddad H, Mendez-Villanueva A, et al Mechanical determinants of acceleration and maximal sprinting speed in highly trained young soccer players. J Sports Sci. 2014;32(20):1906–13. doi: 10.1080/02640414.2014.965191 25356503 10.1080/02640414.2014.965191

[23] Nagahara R, Botter A, Rejc E, Koido M, Shimizu T, Samozino P, et al Concurrent validity of GPS for deriving mechanical properties of sprint acceleration. Int J Sports Physiol Perform. 2017;12(1):129–32. doi: 10.1123/ijspp.2015-0566 27002693 10.1123/ijspp.2015-0566

[24] Stelzer A, Pourvoyeur K, Fischer A. Concept and application of LPM—A novel 3-D local position measurement system. Ieee T Microw Theory. 2004;52(12):2664–9.

[25] Hoppe MW, Freiwald J, Baumgart C, Born DP, Reed JL, Sperlich B. Relationship between core strength and key variables of performance in elite rink hockey players. J Sports Med Phys Fitness. 2015;55(3):150–7. 25069961

[26] Coutts AJ, Duffield R. Validity and reliability of GPS devices for measuring movement demands of team sports. J Sci Med Sport. 2010;13(1):133–5. doi: 10.1016/j.jsams.2008.09.015 19054711 10.1016/j.jsams.2008.09.015

[27] Jennings D, Cormack S, Coutts AJ, Boyd L, Aughey RJ. The validity and reliability of GPS units for measuring distance in team sport specific running patterns. Int J Sports Physiol Perform. 2010;5(3):328–41. 20861523 10.1123/ijspp.5.3.328

[28] Castagana C, Castellini E. Vertical jump performance in Italian male and female national team soccer players. J Strength Cond Res. 2013;27(4):1156–61. doi: 10.1519/JSC.0b013e3182610999 22692110 10.1519/JSC.0b013e3182610999

[29] Michalsik LB, Madsen K, Aagaard P. Physiological capacity and physical testing in male elite team handball. J Sports Med Phys Fitness. 2015;55(5):415–29. 24402441

[30] Hoppe MW, Baumgart C, Hilberg T, Freiwald J, Wehmeier UF. Changes of standard physiological-perceptual markers and circulating MicroRNAs in response to tennis match-play: A case report of two elite players. J Hum Kinet. 2016;51:71–81. doi: 10.1515/hukin-2015-0172 28149370 10.1515/hukin-2015-0172 PMC5260552

[31] EXELIO. GPEXE Pro system technical specifications Udine: EXELIO SRL; 2016.

[32] Catapult. Sprint Help. Melbourne: Catapult-Sports; 2013.

[33] Dellaserra CL, Gao Y, Ransdell L. Use of integrated technology in team sports: a review of opportunities, challenges, and future directions for athletes. J Strength Cond Res. 2014;28(2):556–73. doi: 10.1519/JSC.0b013e3182a952fb 24263650 10.1519/JSC.0b013e3182a952fb

[34] Duffield R, Reid M, Baker J, Spratford W. Accuracy and reliability of GPS devices for measurement of movement patterns in confined spaces for court-based sports. J Sci Med Sport. 2010;13(5):523–5. doi: 10.1016/j.jsams.2009.07.003 19853507 10.1016/j.jsams.2009.07.003

[35] Kinexon. Real-time athlete tracking—Precise. Simple. Smart Munich: Kinexon Precision Technologies; 2016.

[36] Hoppe MW, Baumgart C, Freiwald J. Do running activities of adolescent and adult tennis players differ during play? Int J Sports Physiol Perform. 2016;11(6):793–801. doi: 10.1123/ijspp.2015-0141 26694104 10.1123/ijspp.2015-0141

[37] Romero-Franco N, Jimenez-Reyes P, Castano-Zambudio A, Capelo-Ramirez F, Rodriguez-Juan JJ, Gonzalez-Hernandez J, et al Sprint performance and mechanical outputs computed with an iPhone app: Comparison with existing reference methods. Eur J Sport Sci. 2017;17(4):386–92. doi: 10.1080/17461391.2016.1249031 27806673 10.1080/17461391.2016.1249031

[38] Nagahara R, Morin JB, Koido M. Impairment of sprint mechanical properties in an actual soccer match: A pilot study. Int J Sports Physiol Perform. 2016;11(7):893–8. doi: 10.1123/ijspp.2015-0567 26791405 10.1123/ijspp.2015-0567

[39] Bezodis NE, Salo AI, Trewartha G. Measurement error in estimates of sprint velocity from a laser displacement measurement device. Int J Sports Med. 2012;33(6):439–44. doi: 10.1055/s-0031-1301313 22450882 10.1055/s-0031-1301313

[40] Hopkins WG. Spreadsheets for analysis of validity and reliability. Sportscience 2015;19:36–42.

[41] Hopkins WG. A spreadsheet for combining outcomes from several subject groups. Sportscience 2006;10:50–3.

[42] Stolen T, Chamari K, Castagna C, Wisloff U. Physiology of soccer: An update. Sports Med. 2005;35(6):501–36. 15974635 10.2165/00007256-200535060-00004

[43] Granatelli G, Gabbett TJ, Briotti G, Padulo J, Buglione A, D'Ottavio S, et al Match analysis and temporal patterns of fatigue in rugby sevens. J Strength Cond Res. 2014;28(3):728–34. doi: 10.1519/JSC.0b013e31829d23c3 23722109 10.1519/JSC.0b013e31829d23c3

[44] Ben Abdelkrim N, Castagna C, El Fazaa S, El Ati J. The effect of players' standard and tactical strategy on game demands in men's basketball. J Strength Cond Res. 2010;24(10):2652–62. doi: 10.1519/JSC.0b013e3181e2e0a3 20885192 10.1519/JSC.0b013e3181e2e0a3

[45] Lythe J, Kilding AE. Physical demands and physiological responses during elite field hockey. Int J Sports Med. 2011;32(7):523–8. doi: 10.1055/s-0031-1273710 21563026 10.1055/s-0031-1273710

[46] Kenney WL, Wilmore JH, Costill DL. Physiology of sport and exercise Champaign: Human Kinetics; 2015.

[47] Castellano J, Casamichana D, Calleja-Gonzalez J, Roman JS, Ostojic SM. Reliability and accuracy of 10 Hz GPS devices for short-distance exercise. J Sports Sci Med. 2011;10(1):233–4. 24137056 PMC3737891

[48] Varley MC, Fairweather IH, Aughey RJ. Validity and reliability of GPS for measuring instantaneous velocity during acceleration, deceleration, and constant motion. J Sports Sci. 2012;30(2):121–7. doi: 10.1080/02640414.2011.627941 22122431 10.1080/02640414.2011.627941

[49] Polglaze T, Dawson B, Peeling P. Gold Standard or fool's gold? The efficacy of displacement variables as indicators of energy expenditure in team sports. Sports Med. 2016;46(5):657–70. doi: 10.1007/s40279-015-0449-x 26643522 10.1007/s40279-015-0449-x

[50] Polglaze T, Dawson B, Hiscock DJ, Peeling P. A comparative analysis of accelerometer and time-motion data in elite men's hockey training and competition. Int J Sports Physiol Perform. 2015;10(4):446–51. doi: 10.1123/ijspp.2014-0233 25364940 10.1123/ijspp.2014-0233

[51] Larsson P. Global positioning system and sport-specific testing. Sports Med. 2003;33(15):1093–101. 14719979 10.2165/00007256-200333150-00002

[52] Association FIdF. Approval of electronic performance and tracking system (EPTS) devices Zurich: Federation Internationale de Football Association; 2015.

[53] Akenhead R, French D, Thompson KG, Hayes PR. The acceleration dependent validity and reliability of 10 Hz GPS. J Sci Med Sport. 2014;17(5):562–6. doi: 10.1016/j.jsams.2013.08.005 24041579 10.1016/j.jsams.2013.08.005

[54] Wundersitz DW, Gastin PB, Robertson S, Davey PC, Netto KJ. Validation of a trunk-mounted accelerometer to measure peak impacts during team sport movements. Int J Sports Med. 2015;36(9):742–6. doi: 10.1055/s-0035-1547265 25806591 10.1055/s-0035-1547265

